# Building a Medicinal Chemistry Framework for Bioorthogonal Probes

**DOI:** 10.1021/acscentsci.5c00917

**Published:** 2025-06-17

**Authors:** Markus Staudt, Jonathan C. T. Carlson

**Affiliations:** † Center for Systems Biology, Massachusetts General Hospital Research Institute, Boston, Massachusetts 02114, United States; # Cancer Center, Massachusetts General Hospital Research Institute, Boston, Massachusetts 02114, United States; ‡ Department of Drug Design and Pharmacology, Faculty of Health and Medical Sciences, University of Copenhagen, 2100 Copenhagen, Denmark; § Institute of Applied Synthetic Chemistry, TU Wien, 1060 Vienna, Austria

## Abstract

Screening the tetrazine-protein interactome finds the balance needed for high-performance fluorescent probes.

## Graphical abstract

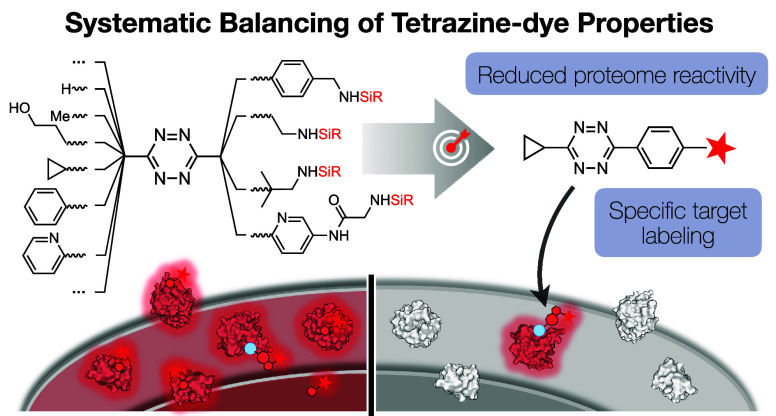

The task assigned to a bioorthogonal probe bears a striking resemblance to that of an irreversible inhibitor: safely traverse an entire ecosystem of biochemical distractions to arrive at a covalent connection in exactly the right niche. Avoid inhospitable, metabolically hazardous terrain; resist lingering in a myriad of tempting biological nooks and crannies. Prevent a spring-loaded connector—be it protein-targeting or click-reactive—from succumbing to the effective molarities of the functional groups encountered along the way.

Tuning the tropism of a complex molecular scaffold to achieve these objectives is the essential work of medicinal chemistry, with ongoing innovations driving active progress in covalent drug discovery.^1^ As we move from an era of bioorthogonal proofs of concept to one of complex multifunctional applications *in vivo*, developing a systematic approach to “bioorthogonal MedChem” will be essential to making the leap to high performance tools. In this issue of *ACS Central Science*, Park, Kim, Lee, and co-workers explore that challenge for a silicon rhodamine tetrazine (Tz) probe.^2^ This is a timely report, coinciding with recent efforts to rigorously map the state of the art in bioorthogonality^3^ or lack thereof,^4^ and paralleling other high impact work to optimize the physiologic stability of bioorthogonal probes.^5^

Beginning with their observation that tetrazine-fluorophore conjugates exhibited unexpected degrees of nonspecific labeling, the groups of Park, Kim, and Lee embarked on a thorough study of the off-target and on-target dynamics of these probes. Mechanistically, the work makes a significant contribution to the understanding of undesired Tz-mediated covalent adduct formation to proteins in the living, cellular context. A combination of model reactions and clever blocking experiments revealed reactivity toward nucleophilic amino acids like lysine and cysteine that have long been suspected but rarely investigated. Screening three different fluorophore-Tz conjugates verified that all three exhibited diverse cell lysate reactivity, each with a distinctive fingerprint on protein gel electrophoresis (Figure 1A).

**Figure 1:**
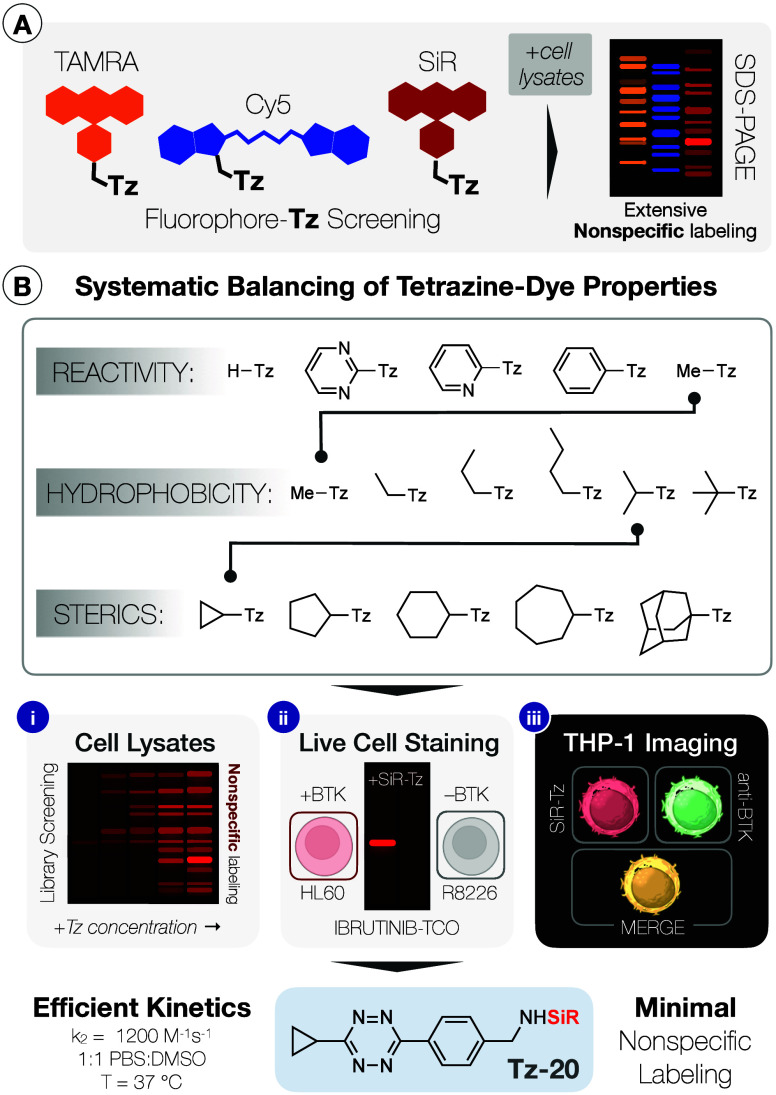
A) Optimally bioorthogonal tetrazine probes should only react with their target dienophiles; here, three different fluorophore-Tz nevertheless exhibited substantial nonspecific binding to native HeLa cell lysates. B) Systematic mapping of the SiR-Tz interactome with escalating stringency: (i) in lysates, as a function of Tz concentration; (ii) in living cells, with an SDS-PAGE readout; and (iii) in live THP-1 cellular imaging with secondary immunofluorescence colocalization. Emerging from this systematic study was the cyclopropane-substituted **Tz-20**, with a winning balance of reaction kinetics and resistance to nonspecific binding that enabled efficient on-target labeling.

Beginning with their observation that tetrazine-fluorophore conjugates exhibited unexpected degrees of nonspecific labeling, the groups of Park, Kim, and Lee embarked on a thorough study of the off-target and on-target dynamics of these probes.

Resisting the easy route of evaluating other commercially available dyes, the team focused on their silicon rhodamine (SiR) probe and prepared a library of more than 20 distinct tetrazines (Figure 1B). This allowed for identification of SiR-Tz with significantly reduced off-target labeling, while also providing a roadmap of the vulnerabilities of these important bioorthogonal tools. Well-known relationships between steric hindrance, electronics, and Tz stability are evident,^6^ but a strong impact of hydrophobicity on protein binding also emerges, with larger/longer hydrophobic substituents exacerbating protein-adduct formation. A cyclopropyl substituent paired with a classic benzylamino Tz was found to provide the best compromise between reaction kinetics, efficient *trans*-cyclooctene (TCO) targeting, and robustness toward the proteome.

Critically, the authors did not stop at posthoc assays in cell lysates; by incubating live cells first with the Bruton’s tyrosine kinase (BTK) inhibitor ibrutinib-TCO, then the SiR-Tz, they established an SDS-PAGE readout for targeting performance within the intact, living cytoplasm. Microscopy studies validated those results, with particularly clean BTK colocalization observed for the winning cyclopropyl **Tz-20** in THP-1 cells (Figure 1B). Splenocyte assays explored by the team were also successful, but less decisive; understanding this difference will be a good topic for future research.

Many other options to build on this insightful work come immediately to mind. An experiment that uses the tetrazines themselves as blocking reagents (sans fluorophore) would be an intriguing addition, helping to highlight the subset of the covalent interactome driven most strongly by the Tz. Likewise, although the Tz moiety mediates the covalent off-target labeling, the protein-specific tropism is strongly influenced by the attached dye. Interestingly, the authors selected a methyl-substituted silicon rhodamine (SiR-Me), perturbing the otherwise close structural similarity to TAMRA. This SiR-Me dye has a known predisposition to mitochondrial localization in other probes,^7^ making it a somewhat unexpected choice here, as the net positive charge may influence both subcellular distribution and washing kinetics. It will thus be highly relevant to see how the cyclopropyl-Tz fares in the habitats visited by other probes with different physicochemical properties.

Going forward, it will thus be essential to develop more general methods to characterize the proteome-dynamics of bioorthogonal molecules that do not incorporate the convenient optical readout of a dye.

More broadly, the propensity of *fluorophore*-Tz probes to react with the proteome mapped here does not speak to the proteome reactivity of *other* conjugates for these same tetrazines, nor does it provide a complete picture of the intrinsic reactivity of the tetrazines themselves. Going forward, it will thus be essential to develop more general methods to characterize the proteome-dynamics of bioorthogonal molecules that do not incorporate the convenient optical readout of a dye. To that end, one alternative strategy could be to equip libraries of new reagents with highly stable minimalist tags like alkynes, providing a handle for later functionalization. Another would be to bring modern high throughput proteomics to bear on this agenda, moving from the largely qualitative nature of SDS-PAGE to mapping events to specific proteins, peptides, and subcellular environments.

The work of Park, Kim, and Lee and their team in this issue of *ACS Central Science* establishes a precedent for systematic screening of fluorophore-Tz probes. Their demonstration of a toolkit-based structure–activity approach to bioorthogonal chemistry arrives at a time when its growing translational potential has fueled increasing interest from both academia and industry. Recent advances such as the rapid access to triazolyl-Tz through copper-catalyzed azide/alkyne click chemistry will facilitate late-stage diversification of scaffolds.^8^ Detailed metabolic screening and custom library synthesis have driven efforts to achieve higher contrast in pretargeted Tz/TCO PET imaging, whether optimizing intravascular reactivity^6^ or tracking the penetration of antisense oligonucleotides beyond the blood–brain barrier.^5^ Finally, the first clinical trials of bioorthogonal therapeutics in cancer patients are now underway, applying the power of biomolecular on/off control to enable new forms of tumor targeting and on-demand prodrug activation.^9,10^

Systematic efforts will be necessary to achieve the pharmacokinetics, metabolic stability, and biodistribution needed for efficient deployment of bioorthogonal tools in the diverse contexts of in vivo settings.

Systematic efforts will be necessary to achieve the pharmacokinetics, metabolic stability, and biodistribution needed for efficient deployment of bioorthogonal tools in the diverse contexts of in vivo settings. Precise control of these factors will be key to translating the unique potential of bioorthogonal chemistry from laboratory into the clinic; the advance from trial-and-error to a medicinal chemistry-like framework for bioorthogonal reactivity profiling is now underway.
